# Investigation of the Bonding Mechanism between Overlapping Textile Layers for FRP Repair Based on Dry Textile Patches

**DOI:** 10.3390/ma16134680

**Published:** 2023-06-28

**Authors:** David Rabe, Juan Daniel Ortega Arbulu, Eric Häntzsche, Chokri Cherif

**Affiliations:** Institute of Textile Machinery and High Performance Material Technology (ITM), TUD Dresden University of Technology, Helmholtzstr. 5, 01069 Dresden, Germany; juan.ortega@tu-dresden.de (J.D.O.A.); eric.haentzsche@tu-dresden.de (E.H.); chokri.cherif@tu-dresden.de (C.C.)

**Keywords:** FRP, novel method repair, dry textile patch, thermoset composites, UV-based depolymerization

## Abstract

Lots of damaged fiber-reinforced plastic (FRP) components are replaced by new components instead of repairing. Furthermore, only very labor-intensive repair methods are available on the market to fully restore the integrity of the structure. This requires a high level of experience or, alternatively, very cost-intensive technology, such as the use of computer tomography and robotics. The high costs and CO_2_ emissions caused by the manufacture of FRP components then bear no relation to their service life. The research project IGF-21985 BR “FRP-Repair” aims to solve the named challenges. Using semiconductor oxide catalysts, the matrix can be locally depolymerized by ultraviolet (UV) radiation, and thus removed from the damaged area of the FRP component. Subsequently, the damaged fibers in this area can be detached. By using customized textile repair patches and local thermoset reinfiltration, the repair area is restored. With this process, the fiber structure can be repaired locally with new fibers on the textile level. The repair is similar to the original production of a fiber composite in an infusion process. No additional adhesive material is used. As a result, repaired FRP structures with restored mechanics and a near-original surface can be realized. This article provides an insight into the actual steps of the development of the FRP component repair process using dry textile patches. The empirical investigation of overlapped rovings and UD material showed the expected results. Residual fracture forces of up to 86% could be achieved. The most interesting approach on the roving level was splicing the overlapping fibers. The free ends of the fibers of the patch and part are mechanically bonded. This bond at the textile level is further strengthened by infusion with matrix.

## 1. Introduction

Fiber-reinforced plastics (FRPs) offer a high lightweight design potential due to their specifically high mechanical characteristics, which is why they are widely used in many industries, e.g., automotive, wind energy, civil engineering, sports and leisure [1,2,3,4]. FRP components usually have high production costs and restricted recyclability and, above all, poor repair opportunities [1,2,5]. 

Previous methods for repairing FRPs are mainly based on shape cutting (e.g., milling) the damaged composite area, the comprising fibers and the matrix. The repair site of the FRP component prepared in this way is then mostly filled up with preimpregnated layers, so-called prepregs [6,7,8,9]. There are several approaches to repair FRP structures, e.g., the scarf method or doubler method [6,10,11,12,13,14,15,16]. These initial repair processes are always associated with high manual effort and manufacturing expenses and often significantly reduce the composite strength of the repaired component or cause extra weight. There is no established procedure to repair FRP components on the textile level by bonding fibers on the textile level. In most cases, complete parts or components have to be replaced [17,18]. End of life (EOL) before the design-based service life leads to a reduction in the environmental and economic profitability of FRP use and to a decrease in lightweight potential. It also significantly reduces the economic and ecological efficiency of FRP production, which is already very energy-intensive.

Recent research efforts [19,20] focused on the development of repair processes specially adapted to FRP. Therefore, the damaged fiber scaffold of the composite parts has to be fixed with new fibers in a different way to state-of-the-art procedures because, normally, the repair procedure is not basically carried out at the composite in total and is not focused on the textile scaffold. To be able to fix the fibers properly by reconnecting to new fibers, the matrix is removed by an oxide semiconductor-supported matrix degradation. By an ultraviolet (UV)-radiation-initialized degradation mechanism, or so-called depolymerization [20], the fibers are detached from the surrounding polymer material to be repaired.

The process starts at the first reinforcement layer of the FRP component. By UV radiation, the matrix is removed. The fibers of the first layer are removed in a specific length to get access to the layers below. After that, the procedure is repeated with all subsequent layers in the direction of thickness until the depth of the damage is reached. Repairable structural damage can be, e.g., structural fracture, delamination or holes. Defects are determined by visual inspection and, if necessary, by nondestructive testing methods. Some defects do not cover the whole cross-section of the structure. Then, the depth of the repair is adapted. The prepared repair area is filled with fitting textile patches in the prepared gradation. With the help of textile processes, called splicing, the patch fibers are connected on the textile level to their counterparts on the boundary of the damage area. The repair area is reinfiltrated with thermoset matrix to restore the composite. The surface can be smoothed afterwards to get back to the former surface and surface quality.

This article gives a brief overview on the repair effect and the benefits of using dry textile patches for damaged composite parts. In this state of the research, a precondition was used to have an idealized material setup, which makes the results comparable. Therefore patch and parent material were combined in a dry textile state and infused together. Particular attention was paid to the interaction between the textile patch and the composite part in the overlap area and the achievable load transfer or regained structural strength after repair.

## 2. Experimental Materials and Methods

### 2.1. Experimental Materials

#### 2.1.1. Raw Materials

Within the study, two different types of carbon fibers were used: T700S-roving material (TORAYCA) and a unidirectional noncrimp fabric (UD-NCF) (Faserverbundwerkstoffe Composite Technology, Waldenbuch, Germany) with PX35 (ZOLTEK). Both fiber types are high-tenacity fibers (HT) with the specific mechanical values summarized in Table 1.

The standard resin for producing the specimens was RIMR 135 mixed with the curing agent RIMH 137 (Hexion) with a 100:30 ± 2 resin-to-hardener mixing ratio (by weight). In a comparing test series, a second epoxy matrix system (-Sika) was used: Sika Biresin^®^ CR83 with the hardener CH83-10 in a mixing ratio of 100:30 by weight as well. In this way, the influence of higher mechanical properties of the second resin system (-Sika) on the strength of the repair were analyzed. The mechanical properties of the pure resin systems are listed in Table 2. Prior to each infiltration process, the resin-hardener mixture was subsequently degassed in a degassing pressure cooker Walther Pilot MDG 12 HZM.

#### 2.1.2. Sample Preparation

Within the project, FRP samples, in this work specifically carbon-fiber-reinforced plastic (CFRP) samples, were used to examine a repair process for FRP on the textile level. The main objective of this study was to analyze and compare the effectiveness of FRP repair based on dry textile patches. The starting precondition for this investigation is an idealized material setup, meaning that the component “to be repaired” is not subjected the previously described procedure, i.e., identifying the size of the damage, removing the matrix out of the damaged area by the UV radiation process, preparing the fiber ends of the parent material, overlapping the fiber ends, reinfiltrating new resin and curing the area. This is necessary because, in former investigations [19], it has been found that many interfering influences come into the process at early stages. Instead of this influential process chain, a cocuring process was used, where both parent material and the repair patch were fitted together in their dry fabric state and manufactured as one component. For the investigation of the performance of the repair method, this workaround is chosen. In further steps within the project, the full repair process is investigated.

The empiric investigation was planned and performed in a rising material complexity. The CFRP specimens were divided into two categories: “Composite A—tow” (CA) and “Composite B—single layered” (CB). While in the CA series, T700S (TORAYCA) carbon fibers were used, for the CB series, a unidirectional noncrimp fabric (UD-NCF) (Faserverbundwerkstoffe Composite Technology) with PX35 (ZOLTEK) carbon fibers was used (cf. Table 1).

**Figure 1:**
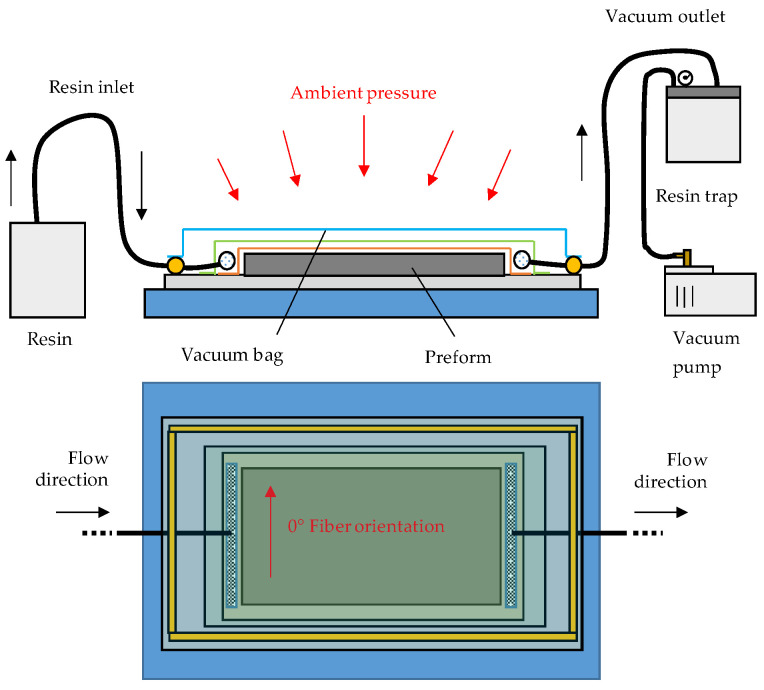
Schematic of VARI manufacturing process: black arrows show the resin flow direction through the setup, red arrows show the ambient pressure compacting the setup.

The fibers were infiltrated with the resin-hardener system (RIMR 135/RIMH 137; SikaCR83/CH83-10) by the vacuum-assisted resin infusion VARI (Figure 1). The VARI manufacturing process is conducted in a closed system (vacuum bag): the depression is generated by a vacuum pump to evacuate the textile preform. The ambient pressure leads the resin to flow into and through the dry carbon fiber fabric (preform) and also to an even compression of the textile preform during the infusion and the subsequent cure. Through prior experimental testing, it was concluded that infiltrating the fibers with resin perpendicular rather than parallel to the fiber orientation resulted in a better resin flow and thus better composite quality. Therefore, the resin inlet and vacuum outlet were positioned as seen in Figure 1.

For the VARI process, a steel plate was used as tool surface. It was cleaned with acetone and then coated with an epoxy mold release agent (Loctite 770-NC). The textile material (preform) was placed on top of the plate and then covered with a perforated PEP foil, which separates the preform from the flow media above. A PES flow channel strip was glued to a PET flow medium next to the preform at the outer edges and then covered with it. Both the flow channel and medium ensure that the resin is properly distributed throughout the entirety of the laminate. The tip of the resin inlet and vacuum outlet were connected and secured to the flow channel. Next, a PA/PE/PA vacuum bag was placed on top of the whole setup and sealed with sealant tape (Tacky Tape SM5142). The air inside was then evacuated and, using a digital pressure gauge, it was verified that it was sealed airtight. Finally, the preform was infiltrated, cured for 15 h at 50 °C on top of a heating plate and then postcured for 15 h at 80 °C in an oven. The different composite configurations investigated in this study had a sample size of at least 5 per series. The WOCO 50 abrasive cutting machine (UNIPREC), with an electroplated diamond cutoff wheel (grit size D427; Pferd), was used to machine the tensile lap-shear test specimens.

#### 2.1.3. Composite A—Overlapping of Tows

**Figure 2:**
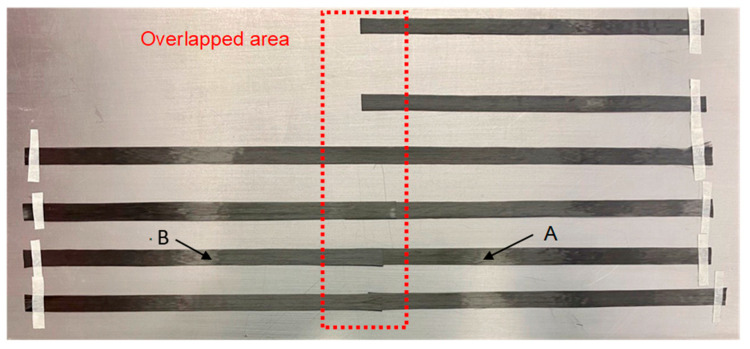
Placing and securing of the overlapped carbon fiber tows A: parent material and B: overlapping ‘patch-material’.

First, examinations were performed on the textile level. Overlapped roving specimens were manufactured for tensile testing and were divided further into 5 subcategories: reference (R), overlap length 1–overlap length 4 (OL1–OL4) and splicing (SPC). R-specimens are made from a continuous 250 mm carbon fiber tow, whereas OL1–OL4 are made from two overlapped 135 mm carbon fiber tows (A and B), as seen in Figure 2. In Table 3, all subcategories of the first series (Composite A—tow) are listed.

Carbon fiber tow A was first placed on the steel plate and secured with an adhesive tape stripe that is placed at the opposite extreme of the overlapped area of the specimen. Then, carbon fiber tow B was placed colinear to A, overlapping the ends at the desired lengths: 5 mm for OL1, 10 mm for OL2, 20 mm for OL3 and 40 mm for OL4 (cf. Table 3). To ensure that A’s and B’s outer edges align with each other during the placing procedure, a steel ruler was aligned and taped down along the edge of A. After matching A and B properly, B was then secured with adhesive tape.

**Figure 3:**
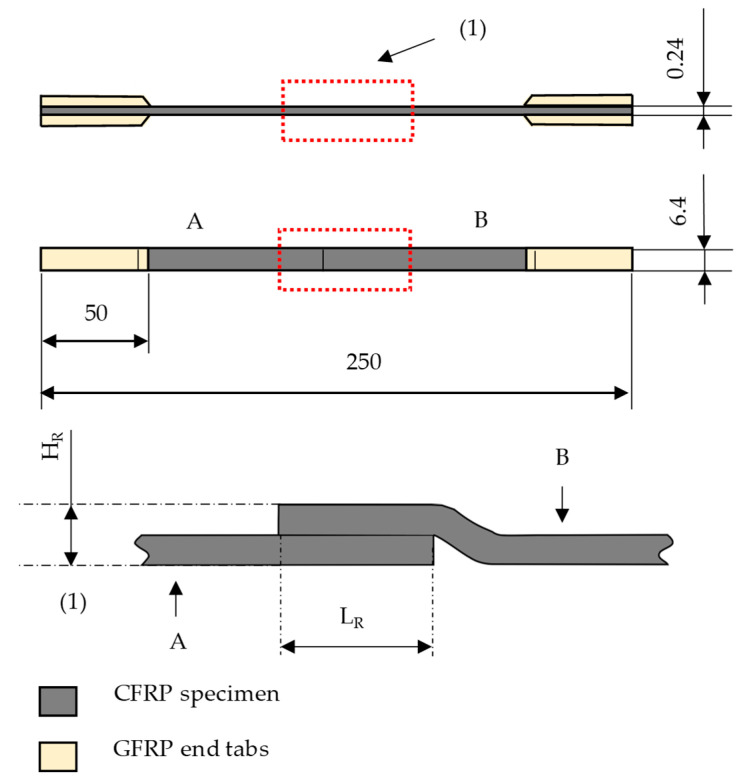
Finished CFRP specimen (OL1, OL2, OL3, OL4): the red dotted square shows the overlapping area which is shown from the top and from the side and is magnified in (1). (1) Magnified side view of the overlap. Repair height, H_R_. Overlap length, L_R_.

Figure 3 shows the finished specimens with OL1-4 and a visual representation of the overlap between A and B viewed from the side. It is imperative to mention that due to the compression generated by the atmospheric pressure during the VARI process, the overlapping fabric (B) must experience some minor bending during the manufacture. Additionally, the overlap generates a local increase in height (H_R_). This is shown in Figure 3.

**Figure 4:**
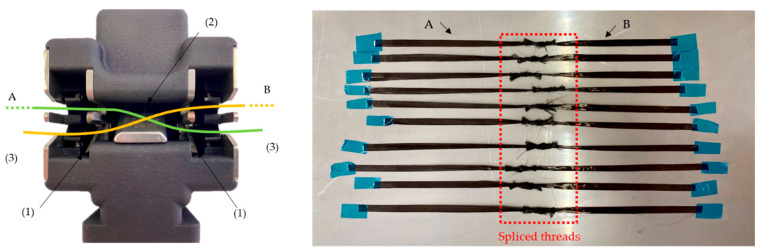
CF thread placement in the air splicer (**left**)—cutting blades (1), splicing chamber (2), excess material (3) of A: green line and B: yellow line. Spliced carbon fiber threads prior to infiltration (**right**).

In addition to the overlapped tow specimens (OL1-OL4, OL3-Sika), spliced (SPC) specimens (250 mm × 6.4 mm × 0.24 mm) of two carbon fiber tows of 150 mm in length were spliced together with an air splicer 141HW (Airbond). For this purpose, both carbon fiber tows (A and B) are placed as seen in Figure 4, on the left. When the device is activated, the splicing chamber (2) closes and flushes pressurized air (4 bar), intertwining both thread ends. The excess material is cut (3). This generates a smooth and strong joint between the carbon fiber threads A and B (Figure 4, right).

Like OL1-4, part A of the specimen was first secured with an adhesive tape on the steel plate, and then B, while simultaneously ensuring for collinearity with a steel ruler. The length of the splice connection between two rovings was about 20 mm. After curing the infiltrated specimens, any excess material was then cut with the WOCO 50 abrasive cutting machine to acquire the target 250 mm specimen length.

#### 2.1.4. Composite B—Single Layered

**Figure 5:**
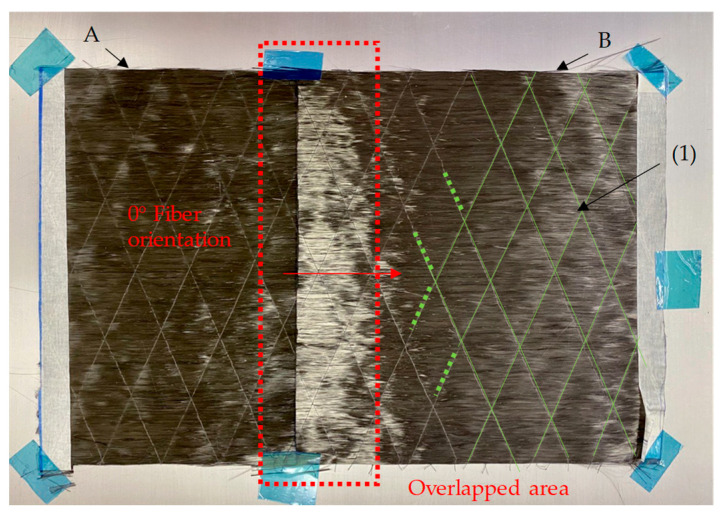
Example of the overlapped CF UD-NCF (N OL3T), polyester adhesive thread (1).

In a second step, specimens from UD material were manufactured for tensile testing and divided in 7 subcategories: reference (R), normal overlap length 1–4 (N OL1–OL4), normal overlap length with thread (N OL3T), modified geometry 1 (M1) and modified geometry 2 (M2). While the R-specimens resulted from a continuous 260 mm × 135 mm × 0.24 mm UD-NCF, N OL1-OL4, N OL3-T and M1-M2 specimens were made from a preform that overlapped two 150 mm × 135 mm × 0.24 mm UD-NCF (Figure 5), following the same principle as performed with the rovings (Composite A). The overlap lengths L_R_ are listed in Table 4. The splicing process as in Composite A—tow was not applied at this stage of the study but will be the subject of future work.

**Figure 6:**
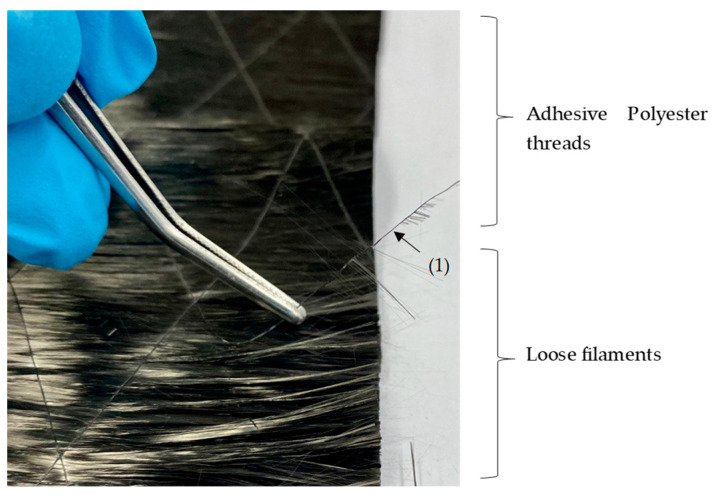
Removal of polyester threads from the CF UD-NCF at the overlapping ends of the fabric. (1) Polyester adhesive thread.

As seen in Figure 5, the UD-NCF is held together with a very thin (76 dtex) adhesive thread grid from polyester (1), which is highlighted in green. These were carefully removed at the overlapped area (cf. Figure 6) for N OL1-OL3 and M1-M2 so that the filaments from B could be compressed more effectively into A during the VARI procedure (Figure 6). To quantify the influence of these polyester threads on the mechanical properties of the composite, they were left attached for N OL3T.

**Figure 7:**
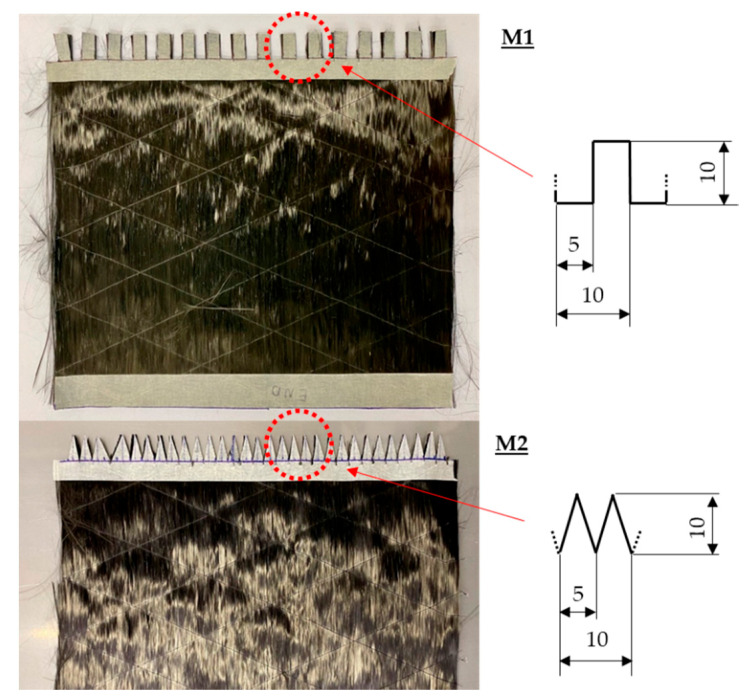
Modified edge geometry of the overlapping fabric B (M1, M2).

To analyze the influence of the edge geometry of the overlapping UD structure (B) (Figure 5), two edge geometry modifications were investigated: one had a rectangle pattern (M1) and the other a triangle pattern (M2) (Figure 7). Adhesive tape was placed on top of the fabric and, with a fine liner, the pattern was marked. Then, the excess material was cut off with microserrated scissors, and the adhesive tape was removed. After removing the polyester thread, the UD structure B was positioned on top of A, as seen in Figure 5. Following the infiltration of the epoxy resin and curing/postcuring of the composite, five specimens were machined (WOCO 50) for each of the configurations for the category “Composite B—single layered”.

### 2.2. Methods

#### 2.2.1. Tensile Testing

**Figure 8:**
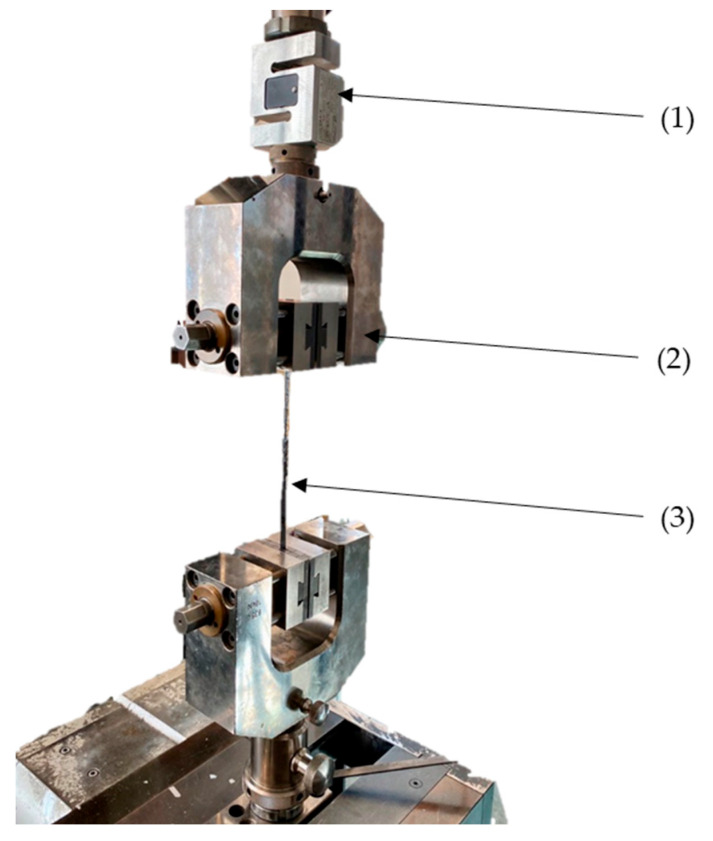
Setup 1 of the tensile testing on the Z100—10 kN force sensor (1), vise tensile grips (2), specimen (3).

**Figure 9:**
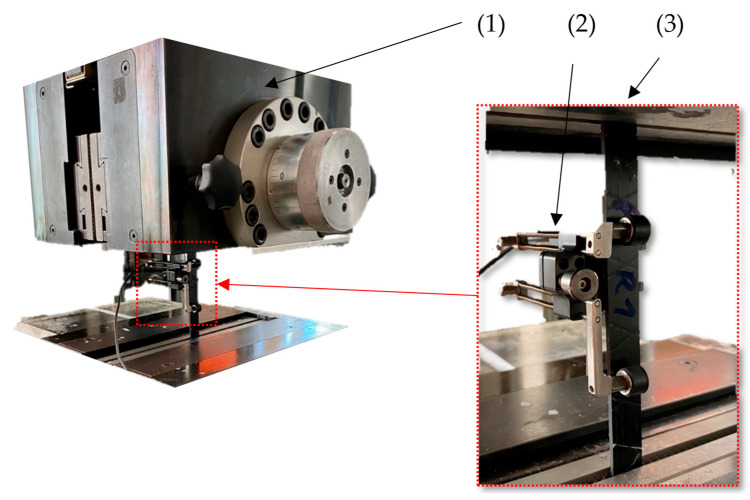
Setup 2 of the tensile testing on the Z100—hydraulic grip (1), clip-on extensometer (2), specimen (3).

The tensile testing (DIN EN ISO 527) [23] was carried out with the universal testing machine Z100 (ZwickRoell, Ulm, Germany). For the specimen of Composite A—tow, a 10 kN force sensor and vise tensile grips were implemented (setup 1, Figure 8). For Composite B (single-layered UD), C (multiple layered) and D (multiple layered 2), the standard force sensor, 100 kN, and hydraulic vice (DEMGEN, profile: sawtooth) were used (setup 2, Figure 9). For all tensile testing specimens, the same machine parameters were used: 150 mm clamping length at start position, 5 N preloading, 2 mm/min testing speed. The young modulus was measured using a clip-on extensometer between 0.05% and 0.25% strain.

#### 2.2.2. Fiber Volume Fraction

The resin burning-off method was used to determine the constituent content of the composite material (CFRP) based on the ASTM D1371. Here, the matrix is physically removed through a heating process up to 625 °C until mass constancy of the sample, leaving the fiber material unaffected. This allows the calculation of the fiber or matrix content by weight and, subsequently, the fiber volume fraction φ of the composite. For that purpose, samples from the different composite configurations (composite A–B) were machined, acclimated, weighted in a mass scale, burned for 1 h at 450 °C and weighted again. Through the residual mass and the known density of the carbon fibers (Table 1) and epoxy resin (Table 1), the fiber volume fraction was calculated thereupon. The following equations were used for the calculation:

$$V_{f}=\frac{m_{r,f}}{\rho_{f}}$$

(1) $$V_{m}=\frac{m_{c^{-m}r,f}}{\rho_{m}}$$

(2) $$\varphi =\frac{V_{f}}{V_{f}+V_{m}}$$

where in the following:

- *m_c_*: Mass of the samples before ignition of the matrix (g).
- *m_r,f_*: Residual mass of the samples (g)
- *ρ_f_*: Density of the carbon fibers (g/cm^3^)
- *ρ_m_*: Density of the matrix (g/cm^3^)
- *V_f_*: Volume of the fibers (cm^3^)
- *V_m_*: Volume of the matrix (cm^3^)
- *φ*: Fiber volume fraction of the composite (%).

## 3. Results

### Tensile Testing

The bonding strength of the prepared overlapped specimen, representing ideally repaired composite structures, was evaluated during tensile testing in two series, each one represented by the type of composite manufactured: Composite A—tow and Composite B—single layered.

**Figure 10:**
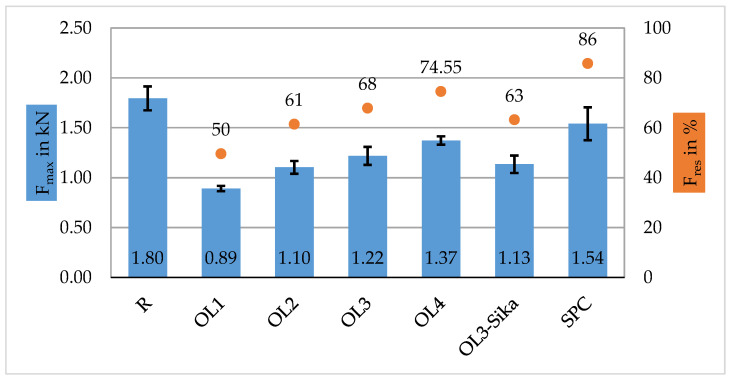
Results of Composite A—tow specimens: Overlap of standard deviation bars marked in red. R: reference. OL1: 5 mm overlap. OL2: 10 mm overlap. OL3: 20 mm overlap. OL3-Sika: with the second matrix system from Sika. OL4: 40 mm. SPC: spliced specimens.

For the first series, Composite A—tow, there is a direct correlation between the length of the overlap and the breaking force of the specimens: the longer the overlap length, the higher the resulting breaking force F_max_ of the specimen (cf. Figure 10). The specimens of the CF-roving (R) infused with reference resin show a breaking force of 1.8 kN. The overlapped specimens show lower breaking forces: OL1 on average 0.9 kN, OL2 on average 1.1 kN, OL3 on average 1.2 kN, OL3 with the second matrix system from Sika 1.1 kN, OL4 1.4 kN and SPC on average 1.5 kN (Figure 10). Relative to the reference specimens (R), this corresponds to the normalized residual strength F_res_ 49.6%, 61.5%, 67.9%, 63.2%, 74.6% and 85.8% of the achievable normalized residual strength (F_res_). The comparison of two different matrix systems leads to the result that the specimen infused with the standard matrix system for this study RimR135/RIMH137 (Hexion) showed around 7% higher breaking forces than the comparative matrix system from Sika.

Regarding the SPC specimens, they were the strongest of all the repaired tows (OL1-OL4), with an ~86% residual strength (F_res_). At the same time, the overlap length was just 20 mm, corresponding with the specimen from OL3. In comparison, an 18% higher residual strength was reached. This is mainly attributed to the mechanical joint in the textile state created by the air splicer (Figure 4), which also confirms the findings in the literature [24], where it has been shown to produce joints on dry yarns with a high residual strength percentage. In future work, the textile splicing connection will be examined in more detail.

**Figure 11:**
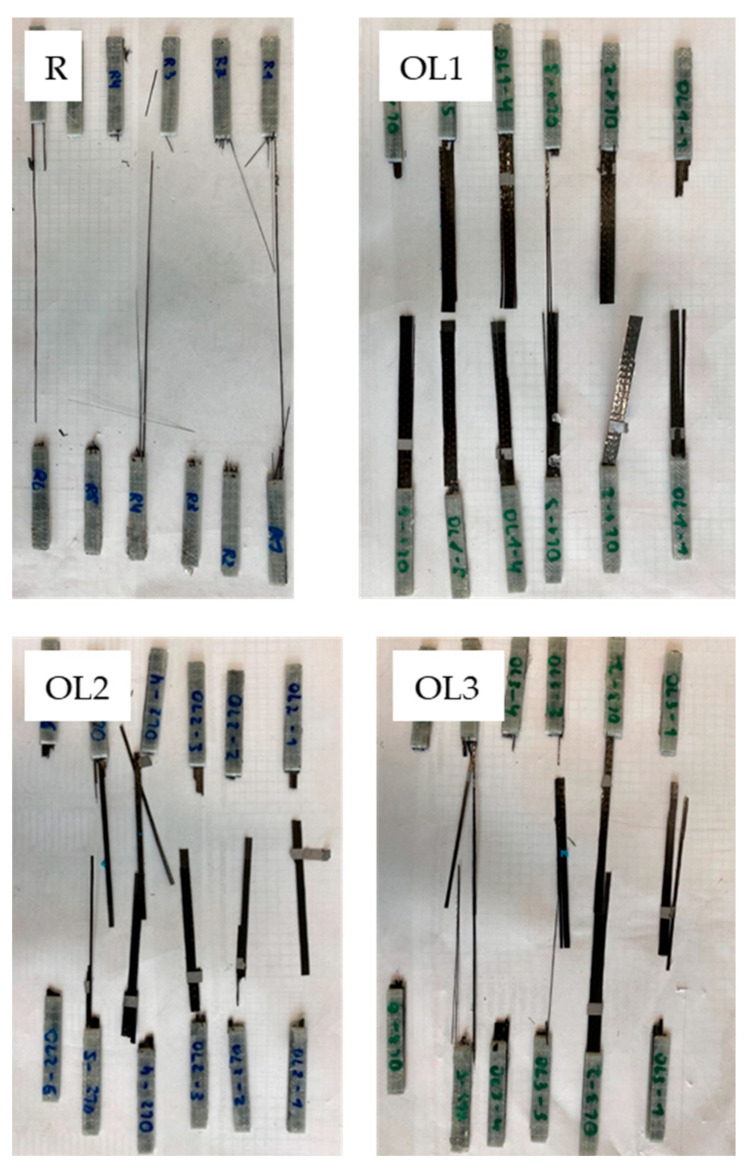
Fracture pattern of the Composite A—tow specimens: R: reference. OL1: 5 mm overlap. OL2: 10 mm overlap. OL3: 20 mm overlap.

The images of the broken specimens (Figure 11, R, OL1–OL3) also reveal that when the overlap length increases, so does the contribution of the fibers to resist against the applied tensile force. The reference samples (R, cf. Figure 11) are completely ‘exploded’, all filaments flew away at the moment of the fracture. The reason for this behavior is the high degree of use of fiber strength. When failing, the sample snaps back, and at the GFRP end taps, there is a second fracture, so that the impregnated roving sample splinters. The higher the degree of fiber strength use, the more the samples splinter. For example, OL1 specimens fractured mainly at the overlap area, leaving the carbon fiber tows relatively unaffected. Thus, the potential strength of the carbon fibers was underused. Meanwhile, OL2 and OL3 show a more emphasized adherend fracture on top of the adhesive fracture, which can be correlated to a higher degree of force transmission through the fibers and therefore a higher resistance against the applied load. The higher the overlap length of the OL specimen (OL1–OL4), the more the broken specimens look like the reference. The fractured spliced samples (SPC) look like the reference; meanwhile, they show the highest breaking forces in between the repair-representing specimen.

**Figure 12:**
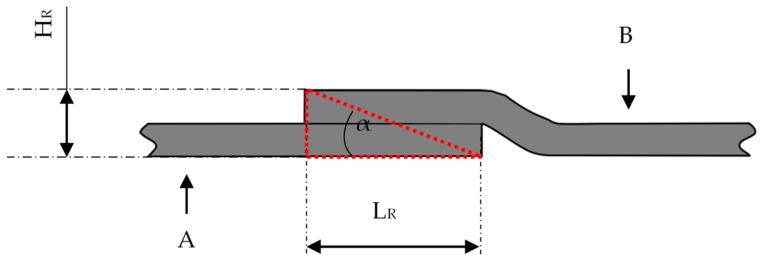
Schematic of the repair for Composite A—tow: repair height *H_R_*, overlap length *L_R_*, scarf angle (α), parent material (A), repair patch (B).

Moreover, it was noticed that within the first series Composite A—tow, the carbon fiber filaments of B (repair patch) are just slightly pressed into the filaments of A (parent material) at the overlap area (repair area). There is no fiber mixing or connection in either the textile or composite state. Therefore, the repair area can be treated as a step overlapped joint, and a scarf angle (cf. Equation (3)) can be defined, as seen in Figure 12. The repair height of the Composite A—tow specimen was 0.3 mm, and the overlap length varied for the different overlap lengths.

(3) $$\alpha =arctan\left(\frac{H_{R}}{L_{R}}\right)$$

The notation in Equation (3) is defined as follows:

- α: Scarf angle (°).
- *H_R_*: Composite height at the repair area (mm).
- *L_R_*: Composite length of the repair area (mm).

This results in scarf angles of 3.44° for OL1, 1.72° for OL2, 0.86° for OL3 and 0.43° for OL4. A strong CFRP joint and CFRP repair patch should strive to have a scarf angle of α ≈ 2°, and the closer the scarf angle approaches the value of 1 °, the less noticeable the improvement of the bonding strength. This further amplifies the tensile testing results that OL2 was ~ 12 % stronger than OL1, but OL3 was only ~6% stronger than OL2. In addition, the standard deviation of OL2 and OL3 overlap (Figure 10) indicates that the difference between both series is not statistically significant.

**Figure 13:**
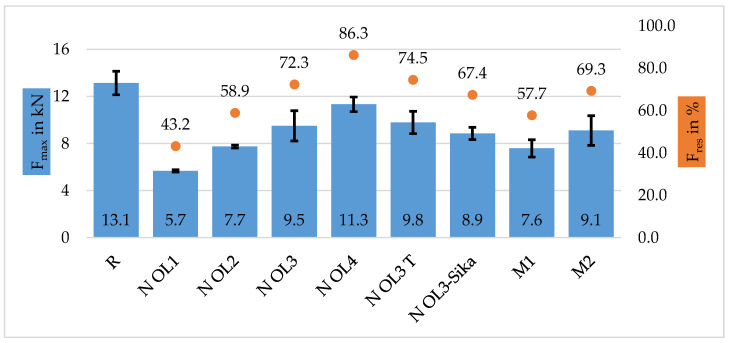
Results of Composite B—single layered: R: reference. N OL1: 5 mm overlap. N OL2: 10 mm overlap. N OL3: 20 mm overlap. N OL3T: 20 mm overlap with thread. OL3-Sika: 20 mm overlap with second matrix system Sika. M1: 20 mm overlap with modified edge geometry (Type 1). M2: 20 mm overlap with modified geometry (Type 2). OL4: 40 mm overlap.

The specimens of Composite B (single layered) show similar characteristics, shown in Figure 13 observed in the first series: Composite A—tow. The breaking force of the repaired specimen also increase with greater overlap length, where N OL1 (LR = 5 mm) had a residual strength of 43.2%, N OL2 (LR = 10 mm) 58.9%, N OL3 (LR = 20 mm) 72.3% and N OL4 (LR = 40 mm) 86.3%. However, for this series, the difference in performance is more prominent than in the series of Composite A.

**Figure 14:**
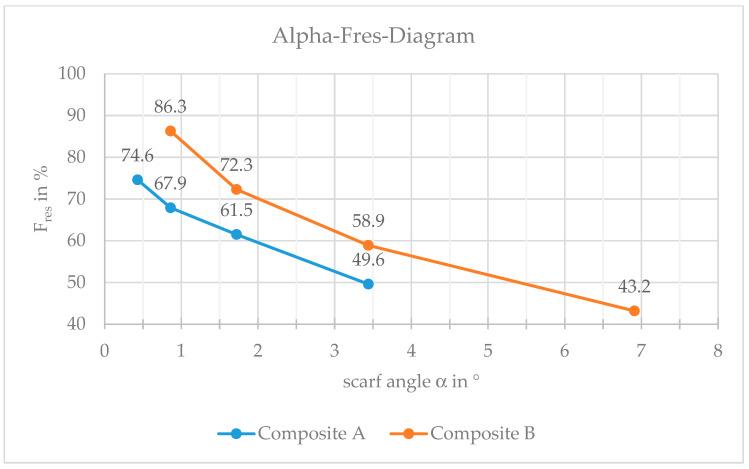
Relationship between scarf angle and residual breaking force of the repaired specimens: Composite A (blue) and Composite B (orange). Fres: residual breaking force. α: scarf angle.

One possible reason becomes evident after calculating the scarf angles: 6.91° (N OL1), 3.44° (N OL2), 1.72° (N OL3) and 0.86° (N OL4). The resulting scarf angle varies because of the thickness of the raw material (UD-NCF) and, following the repair height of Composite B (0.6 mm), it is thicker than in Composite A. The scarf angle is dependent of the length and the height of the repair area. Figure 14 depicts the residual strength of both composites A and B as a function of the scarf angle, where the lower the scarf angle, the higher the residual breaking force of the specimen.

When comparing the specimens of N OL3 and N OL3T (LR = 20 mm, polyester thread left attached), leaving the polyester thread shows a slight improvement in residual strength: 72.3% for the former and 74.5% for the latter. Nevertheless, upon analyzing the standard deviations, which are overlapping, it is found that there may not be a statistical significance between leaving or removing these threads. After further analyzing the overlap area, many fibers are not aligned properly with the fibers of the parent material because of the removal of the fixing thread. If the fibers are not aligned properly with the *x*-axis of the subsequent composite, the load transmission is not optimal. This deficit can be attributed to the stacking of the workpiece prior to the infiltration of the resin. This factor may explain why the standard deviation of N OL3 is relatively high. But, in general, it is possible to say that there is not that great an influence from fixing thread material in the overlap area in terms of overlapping and contacting fibers ends.

**Figure 15:**
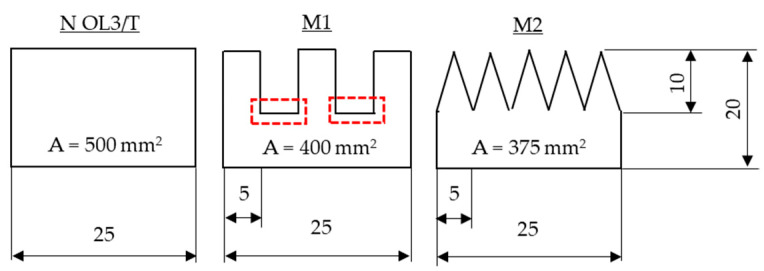
Comparison of the surface area of the overlap for N OL3-T, M1 and M2.

The specimens with modified edge geometry (M1, M2) show a slightly lower residual strength percentage than the specimens without edge geometry modification: 57.7% (M1) and 69.3% (M2). This may be because the surface area of the overlap of the specimens without modification is bigger than the surface area of the specimens with modification (cf. Figure 15). Moreover, despite M2 having a slightly lower surface area, it is about 12% stronger than M1. It can be assumed that due to the two wider stress spike locations (marked in red; Figure 15), the specimens of M1 have a lower breaking force. Another observation is that M1 has similar residual breaking force values to the specimens without edge geometry modification at an overlap length of 10 mm (N OL2). The weakest link of M1 is also located at 10 mm, and possibly, the debonding starts here. Conversely, M2 may have a more gradual and uniform stress distribution throughout the width of the specimen due to its geometry. Nevertheless, an FEM analysis would be necessary to reveal how the stress distributes throughout the repair width.

**Figure 16:**
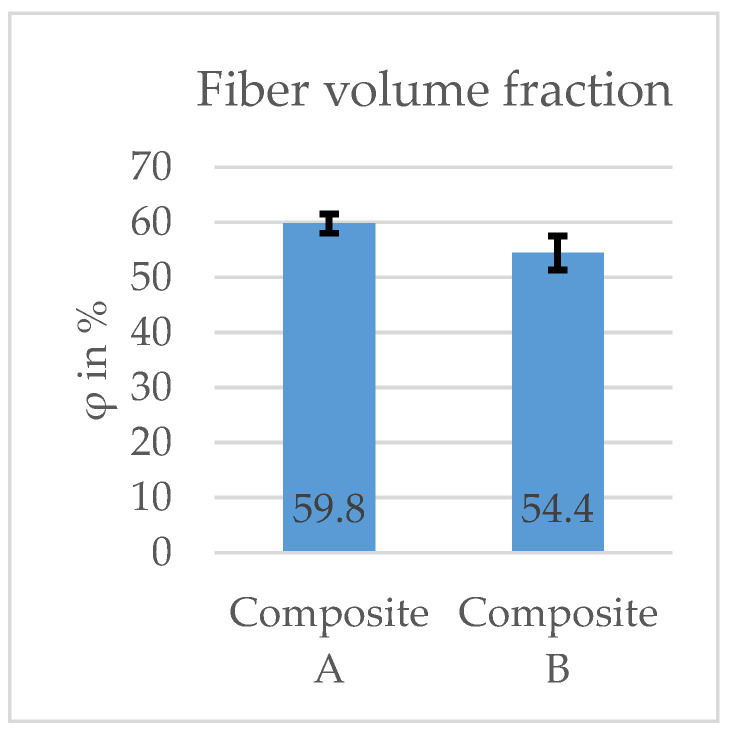
Average fiber volume fraction of the manufactured CFRP workpieces.

The fiber volume fraction is a key property of CFRP components because it is strictly linked to the mechanical properties. After taking samples and implementing the resin burning-off method (cf. Section 2.2.2), the fiber volume fraction φ (cf. Equation (2)) across all samples showed consistent results: 59.8% and 54.4% for composites A and B (Figure 16). Moreover, there were no significant differences in values when the sample was taken outside or inside the repair patch. The VARI processing resulted in high fiber volume fraction in both series. The standard deviation in between the series is reasonable.

## 4. Discussion

### 4.1. Composite A—Tow

The results of the first composite series A—tow are quite interesting in terms of FRP repair at the textile level. The previous approach of simply overlapping the free fiber ends of the patch and the component to be repaired does not work properly. Overlapping the fiber ends in increasing length without mechanically bonding the fibers by mixing is not as strong, and even at a high overlap length of 40 mm, the bond breaks at about 75% of the original breaking strength. The theoretically decreasing scarf angle shows the expected effect, but overall, the residual strength is too low for a solid FRP repair. Not taken into account is the fact that an overlap of 40 mm is not realistic for an ecological and economical repair. For this instance, the repair area must be as small as possible to avoid removing and reapplying a large amount of material. From an economic and ecological view, the overlap of fibers without any mixing of the ends (OL1–OL3) is not appropriate. The resulting strengths are not worth repairing components. The data were obtained to have a reference for future work.

The approach of mechanically splicing the free fiber ends of the patch and the component to be repaired together to achieve a bond at the textile level worked well in this first trial at the roving level. With about 86% residual strength, the sample gave more than 10 % higher values than the samples with 40 mm overlap; considering the spliced joint is only about 20 mm long, the effect of splicing is quite large. Considering this result, future work will focus on this approach to achieve a higher residual strength due to the repair. The results from splicing from the roving level to flat semifinished products will have to be transferred.

Another point of interest was the change in the repair resin system and the effect on repair strength. The sample prepared with a nominally stronger resin system (-Sika) did not guarantee stronger repair adhesion. The resulting values were actually lower with the second matrix system (-Sika). For future work, this means that further tests with different matrix systems have to be carried out to obtain a more comprehensive overview of this issue.

### 4.2. Composite B—Single-Layered

The general results of the Composite A series (tow) could be verified and confirmed with the Composite B series (single layered). With increasing overlap length (5–40 mm) and the associated decreasing scarf angle, the residual breaking force increases. The overlap of 40 mm is investigated only to extend the field of knowledge, as this length is not practical for performing repairs on real structures, as the repair area would become too large. As already mentioned in 4.1, a repair with just overlapping fiber ends (N OL1–N OL3) does not lead to a worthwhile repair result. And the high overlap length of 40 mm (N OL4) leads to a very huge repair area, which makes the repair process less efficient, because a lot of material must be removed and exchanged by patch material and resin.

There is a small difference between the sample with the string removed (N OL3) and the sample with the string remaining on the fibers (N OL3 T). Due to the overlap of the standard deviation, there is no significant difference between removing and leaving the binding thread on the UD sheet.

As for Composite A series, changing the matrix system results in a small decrease in residual breaking strength (N OL3—Sika vs. N OL3).

The M1 and M2 series with a modified overlap area geometry showed lower residual breaking forces compared with N OL3-T. The differences in overlap area only result in lower fracture forces, probably due to local stress concentrations. The approach using the modified geometry of the textile patch geometry is not expedient.

## 5. Conclusions

This paper presents the latest results in the development of a new repair process for FRP, with the specific example of CFRP, at the textile level. For comparability of the results, an ideal starting condition of a co-infusion of patch and parent material was used. The empirical investigation with different overlap lengths and different coupling mechanisms showed the expected results. The overlap of two textile layers in the raw textile state (tow or single-layer material) leads to residual fracture forces of up to 86%, whereas in a realistic and industrial practical overlap length range (up to 20 mm), only 75% of the new value fracture force is achieved. The simple overlap results in a force-fit joint due to load transfer via shear in the epoxy matrix. The approaches of replacing the resin system with a “stronger” one or changing the geometry of the overlap area do not show better results.

The most interesting approach was splicing the overlapping fibers. The end of the patch fiber is mechanically bonded to the free fiber end of the starting material. This bond at the textile level is further strengthened by infusion with the matrix. The connection is thus converted from a force-fit joint only (simple overlap) to a form- and force-fit joint connection. These test specimens result in breaking forces of 86% of the virgin material. These promising results from tow material will be transferred to sheet material in the next step. Further investigations of the connection between patch and base material on the textile and composite level will be carried out in the near future. With the help of this new approach in the repair of FRPs, components can be used more economically and ecologically up to their lifetime limit.

## Figures and Tables

**Table 1:** Specific mechanical values of the used carbon fibers.

Fiber Properties	Torayca T700S	Zoltek PX35
Tensile strength (MPa)	4900	4137
Tensile modulus (GPa)	230	242
Elongation (%)	2.1	1.5
Density (g/cm^3^)	1.80	1.81

**Table 2:** Specific values of the used epoxy resins from the supplier data sheets [21,22].

Mechanical Properties	RIMR135/ RIMH137	CR83/ CH83-10 (-Sika)
Tensile strength (MPa)	60–75	86
Tensile modulus (GPa)	2.7–3.2	3.1
Elongation at break (%)	8–16	7.9
Flexural strength (MPa)	90–120	131
Impact strength (KJ/m^2^)	70–80	83
Compressive strength (MPa)	80–90	109
Density (g/cm^3^)	1.18–1.20	1.15

**Table 3:** Designation of the first series: Composite A—tow.

Subcategory	Overlap Length in mm	Note
OL1	5	-
OL2	10	-
OL3	20	-
OL4	40	-
OL3-Sika	20	Usage of nominal stronger resin system from “Sika”
SPC	~20	Mechanical connection on textile level by splicing with an air splicer

**Table 4:** Designation of the second series: Composite B—single layered.

Subcategory	Overlap length L_R_ in mm	Note
N OL1	5	-
N OL2	10	-
N OL3	20	-
N OL4	40	-
N OL3-Sika	20	Usage of nominal stronger resin system from “Sika”
M1	~20	Modified edge geometry, rectangle pattern
M2	~20	Modified edge geometry, triangle pattern

## Data Availability

The data underlying this article will be shared on reasonable request from the corresponding author.
